# Isn’t it ironic? Functional iron deficiency at the core of Parkinson’s disease pathobiology

**DOI:** 10.1172/JCI202244

**Published:** 2026-01-02

**Authors:** Ian Peikon, Nancy C. Andrews

**Affiliations:** 1 Cajal Therapeutics Inc., Seattle, Washington, USA.; 2 Department of Pediatrics, Boston Children’s Hospital and Harvard Medical School, Boston, Massachusetts, USA.

Aberrant iron homeostasis has been implicated in Parkinson’s disease (PD) for decades. Currently, the dominant view is that iron overload in neurons of the substantia nigra drives pathogenic neurodegeneration. This view is supported by histology, MRI studies, and the discovery of ferroptosis, an iron-dependent form of cell death. However, recent clinical trials using the brain-penetrant iron chelator deferiprone (DFP) have shown that iron removal worsens PD symptoms, particularly in drug-naive patients (1, 2), challenging this model.

An alternative explanation for iron’s role in PD is functional iron deficiency, a state in which the total amount of iron is normal or elevated, but bioavailable ferrous iron (Fe²^+^) is reduced due to sequestration. Functional iron deficiency is well documented in inflammatory conditions (3), and the effects of iron deficiency on iron-dependent processes, including impaired mitochondrial respiration and decreased dopamine synthesis, align closely with PD hallmarks (4). Historical reports suggest that modest iron supplementation can improve PD symptoms (5), likely via stimulation of tyrosine hydroxylase (TH), the iron-dependent and rate-limiting enzyme in endogenous dopamine production. A similar rationale underlies the use of i.v. iron therapy in restless leg syndrome (RLS), another dopaminergic disorder (6).

Taken together, these observations point toward potential clinical benefit from restoring iron bioavailability and harm from limiting it in PD. Here, we detail the data suggesting that functional iron deficiency could be a key mechanism in PD and argue that pursuing this concept could lead to novel therapeutic avenues.

## From l-DOPA to iron therapy

The first breakthrough in PD treatment came in the early 1960s when Birkmayer and Hornykiewicz showed that l-3,4-dioxyphenylalanine (l-DOPA) alleviated motor symptoms caused by dopamine deficiency in the basal ganglia, a group of midbrain nuclei that includes the substantia nigra (5). This approach was later justified by the finding that TH activity, which converts tyrosine to l-DOPA, was diminished in the PD brain (6). It was later shown that TH is an iron-dependent enzyme (7) and that iron could dramatically stimulate TH activity in human brain homogenates (8), prompting Birkmayer to explore iron therapy as a treatment for PD. In the 1980s, Birkmayer reported striking improvements in a study of 100 PD patients given iron supplementation, with some able to stop dopaminergic drugs entirely (9). While this study was not conducted in a controlled clinical trial setting, it nevertheless suggests the potential of iron therapy in PD. It is surprising that no additional work has been done in this vein, especially given that RLS, which is also a dopaminergic disorder, is treated with iron therapy as standard of care (10).

## The rise of the iron overload hypothesis

Despite indications that iron therapy could be beneficial in PD, the field shifted toward the now dominant hypothesis of iron overload in PD pathogenesis. By the late 1980s and the 1990s, histochemical and MRI relaxometry studies had shown evidence of increased iron content in the substantia nigra of PD patients (11). This fueled the hypothesis that excess iron contributes to neurodegeneration via oxidative stress. The concept gained further traction after ferroptosis was described in 2012 (12). Multiple investigator-initiated studies followed to test the effects of DFP, an iron chelator that crosses the blood-brain barrier, in patients with PD. The largest study, completed in 2022 (1) and confirmed in subsequent trials (2), found that iron chelation led to marked symptom worsening in drug-naive PD patients, challenging the iron overload model of disease.

## Iron overload may be an illusion

Iron exists as Fe³^+^ (ferric iron) and Fe²^+^ in biological systems. Fe³^+^ is the predominant inert form and is found bound to transferrin for transport in the blood and between tissues as well as in ferritin cages (~4,500 Fe³^+^ atoms), which store excess iron in cells. Fe²^+^, by contrast, fuels enzymatic catalysis, mitochondrial iron–sulfur (Fe-S) cluster biogenesis, and Fenton chemistry.

MRI is more sensitive to Fe³^+^ because it is more paramagnetic than Fe²^+^. Moreover, Fe³^+^ is more likely to be found at a high concentration due to the natural sequestration of Fe³^+^ in close proximity in ferritin cages and in melanin granules (13). In PD, histological methods (Perls’ staining), MRI, and atomic spectroscopy consistently identify increased Fe³^+^ in the area of the substantia nigra (14, 15). These data were used to support the idea that excess iron was likely driving oxidative stress and that removal of excess iron via chelation would be therapeutic (1). However, Fe²^+^ is responsible for driving oxidative stress, and Fe³^+^ abundance in tissue is a poor proxy for Fe²^+^ status.

Elevated Fe³^+^ may indeed signal an excess of total iron (Fe³^+^ and Fe²^+^), but it can also be a marker of an underlying defect in iron handling that is associated with cellular iron deprivation. For example, in chronic inflammation, macrophages and other cells sequester iron in Fe³^+^ form to prevent iron-hungry pathogens from accessing host iron (3). Anemia of chronic inflammation can thus present with normal or even high tissue iron levels, but with an underlying iron deficiency in red blood cell precursors (3). Lysosomal dysfunction can also lead to accumulation of Fe³^+^ in the endolysosomal compartment, as the release of Fe³^+^ from transferrin and the subsequent reduction of Fe³^+^ to Fe²^+^ for transport into the cytoplasm are both pH-dependent processes (16). When sequestration like this occurs, cells sense persistent Fe²^+^ deficiency and attempt to import more iron (largely via upregulation of the transferrin receptor; ref. 17), which may also become trapped, creating a feed-forward open loop. This is akin to an old house where a thermostat (cytoplasm) and heater (endosome) are in separate rooms with the door shut between them. The thermostat continually senses a cold room and demands more heat, while the heater is running at maximum in the hot room next door.

## Limitations of MRI measurements of tissue iron

Whether iron in PD accumulates within dopaminergic neurons or in other cell types has not been definitively established. Iron could be accumulating in the endolysosomal compartment of the dopaminergic neuron, as described above, leaving iron unavailable for other cellular processes like mitochondrial respiration and dopamine synthesis. Alternatively, iron could be accumulating in other cell types nearby, depriving dopaminergic neurons of iron. In aceruloplasminemia, a parkinsonism caused by loss of ceruloplasmin (an oxidase required for cellular iron export), iron accumulates in the basal ganglia, especially in astrocytes. Data in mouse models of aceruloplasminemia suggest that neurons may be relatively iron starved, at least early in disease (18). Iron could also accumulate in microglia, as in multiple sclerosis, where iron-laden macrophages/microglia form rims at the edges of chronic demyelinated lesions (19). Unfortunately, MRI lacks the spatial resolution to reveal which cell types (or subcellular compartments) drive the observed signal, which could be masking an underlying dopamine neuron iron deficiency.

Recent MRI studies indicate that drug-naive PD patients present with reduced nigral iron at disease onset, that iron accumulates during disease, and that higher iron content is associated with levodopa usage (20). Levodopa and other catechols are iron chelators (21). During treatment, high exogenous levels of levodopa and its catechol metabolites can complex with iron, leading to sequestration within dopaminergic neurons, microglia, or astrocytes. Therefore, bulk iron estimates via MRI may be shaped by disease stage and/or therapeutic exposure, and current data provide insufficient longitudinal insights to draw definitive conclusions about cellular iron status in PD.

**Figure 1:**
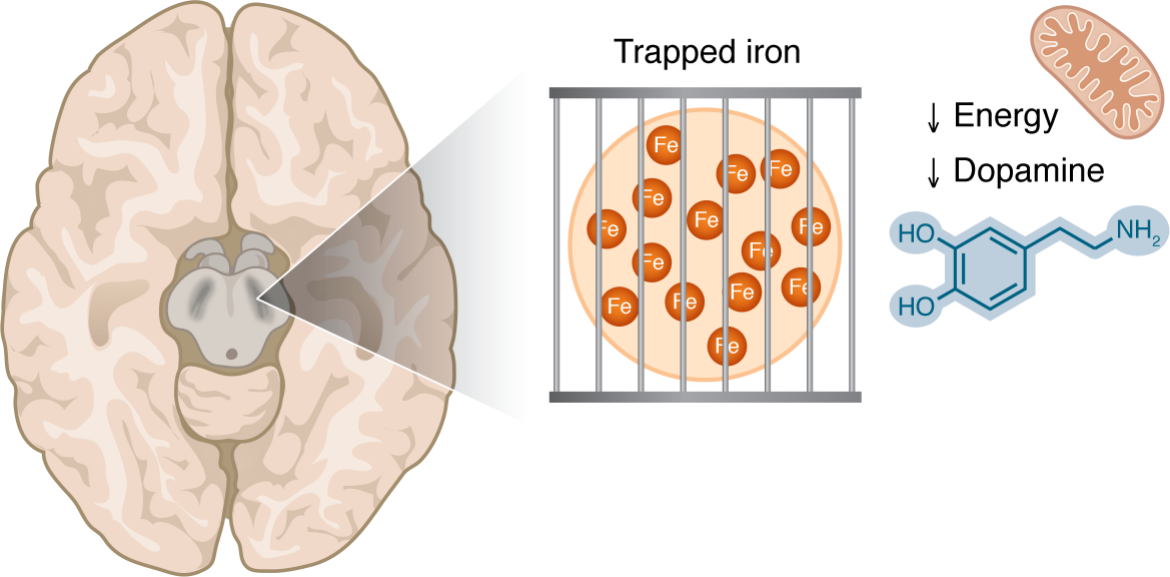
Iron accumulation in the substantia nigra is visible by MRI techniques in patients with PD. This iron may be in a trapped form, making it unavailable for the iron-dependent biological processes that are critical in dopaminergic cells, including mitochondrial respiration and dopamine synthesis.

Together, the shortcomings of MRI-based techniques (lack of ability to measure and distinguish Fe^3+^ and Fe^2+^, lack of cellular and/or subcellular resolution, and paucity of available individual longitudinal data) highlight the need for additional caution when interpreting MRI-based measures of iron. Though it may appear that patient brains are overloaded with iron, the data are equally consistent with a functional iron deficiency of the dopaminergic neurons. While the total amount of iron in the tissue may be normal or elevated, bioavailable Fe²^+^ in the dopaminergic neurons is low due to sequestration in subcellular compartments or nearby cells (Figure 1).

## Evidence for functional iron deficiency in parkinsonism

Further support for the idea that iron deficiency may be at the root of PD comes from studies on manganism, another disorder that phenocopies major components of PD (22). Manganism is caused by high exposure to manganese, which is known to perturb cellular iron homeostasis and produce an iron-deficient cellular phenotype (23). This may occur, in part, because manganese can substitute for iron in several processes, including binding to transferrin and subsequent uptake of manganese–transferrin complexes via receptor-mediated endocytosis. Sustained exposure ultimately decreases mitochondrial aconitase (24) and TH activity (25), two iron-dependent processes critical for mitochondrial respiration and dopamine synthesis, which may partly explain why the phenotypes of manganism overlap with those of PD.

Another group of disorders, collectively termed neurodegeneration with brain iron accumulation (NBIA), also exhibit some phenotypic overlap with PD. NBIA is genetically and clinically heterogeneous, with documented cases that include iron accumulation in the basal ganglia, levodopa-responsive parkinsonism, and synuclein aggregation (26). It is therefore tempting to ask whether NBIA can teach us something about iron in PD pathobiology. One of the most common forms of NBIA results from mutations in *PANK2*, a gene involved in CoA biosynthesis. CoA is required for the function of mitochondrial acyl carrier protein, which plays a central role in Fe-S cluster biogenesis (27). Consequently, *PANK2* mutations are expected to impair Fe-S cluster formation. Failure in Fe-S cluster biogenesis triggers an iron starvation response, driving additional iron import into mitochondria (28), despite its inability to be used for downstream Fe-S cluster biogenesis. This phenomenon mirrors the thermostat analogy described above and suggests that apparent iron overload may be a strong biomarker for defects in the proper usage of iron within the cell.

Finally, and most directly, mouse genetic work has suggested that iron deficiency due to deletion of the transferrin receptor (which imports iron via the transferrin-iron complex) causes loss of dopaminergic neurons in a pattern similar to PD as well as neurobehavioral changes associated with murine parkinsonism (4).

## Rethinking iron’s position in PD pathology and therapy

In conclusion, multiple lines of evidence call into question the iron overload hypothesis in PD pathology. If we consider instead that cells in humans with PD may be suffering from a functional iron deficiency (4), much more of the available data make sense. Epidemiological studies link systemic anemia and recent blood donations to higher PD risk (29). TH function and mitochondrial respiration both rely on iron; thus, functional iron deficiency will lead to decreases in dopamine tone and loss of mitochondrial respiration (which ultimately will drive cell death), both of which are hallmarks of PD. Iron removal via chelation only exacerbates these problems, particularly in drug-naive patients where excess l-DOPA is not on board to mask a decline (1, 2). Iron therapy, in RLS and in PD, benefits patients (9, 10). Removing iron in brains of patients with PD via chelation has been sufficiently tested clinically, with negative effects on patient outcomes. We should now consider the alternative hypothesis of functional iron deficiency and how we might tackle it therapeutically.

## Version 1 01/02/2026

Electronic publication
